# Resistome and a Novel *bla*_NDM-1_-Harboring Plasmid of an *Acinetobacter haemolyticus* Strain from a Children's Hospital in Puebla, Mexico

**DOI:** 10.1089/mdr.2019.0034

**Published:** 2019-09-09

**Authors:** Elena Bello-López, Semiramis Castro-Jaimes, Miguel Ángel Cevallos, Rosa del Carmen Rocha-Gracia, Miguel Castañeda-Lucio, Yolanda Sáenz, Carmen Torres, Zita Gutiérrez-Cazares, Ygnacio Martínez-Laguna, Patricia Lozano-Zarain

**Affiliations:** ^1^ Centro de Investigaciones de Ciencias Microbiológicas, Instituto de Ciencias, Benemérita Universidad Autónoma de Puebla, Puebla, México.; ^2^ Programa de Genómica Evolutiva, Centro de Ciencias Genómicas, Universidad Nacional Autónoma de México, Cuernavaca, México.; ^3^ Área de Microbiología Molecular, Centro de Investigación Biomédica de La Rioja (CIBIR), Logroño, España.; ^4^ Área Bioquímica y Biología Molecular, Universidad de La Rioja, Logroño, España.; ^5^ Hospital para el Niño Poblano, Puebla, México.

**Keywords:** *A. haemolyticus*, plasmid, NDM-1, antibiotic resistance

## Abstract

*Acinetobacter calcoaceticus-baumannii* complex isolates have been frequently associated with hospital and community infections, with *A. baumannii* being the most common. Other *Acinetobacter* spp. not belonging to this complex also cause infections in hospital settings, and the incidence has increased over the past few years. Some species of the *Acinetobacter* genus possess a great diversity of antibiotic resistance mechanisms, such as efflux pumps, porins, and resistance genes that can be acquired and disseminated by mobilizable genetic elements. By means of whole-genome sequencing, we describe in the clinical *Acinetobacter haemolyticus* strain AN54 different mechanisms of resistance that involve *bla*_OXA-265_, *bla*_NDM-1_, *aphA6*, *aac(6’)-Ig*, and a resistance-nodulation-cell division-type efflux pump. This strain carries six plasmids, of which the plasmid pAhaeAN54e contains *bla*_NDM-1_ in a Tn125-like transposon that is truncated at the 3′ end. This strain also has an insertion sequence IS*91* and seven genes encoding hypothetical proteins. The pAhaeAN54e plasmid is nontypable and different from other plasmids carrying *bla*_NDM-1_ that have been reported in Mexico and other countries. The presence of these kinds of plasmids in an opportunistic pathogen such as *A. haemolyticus* highlights the role that these plasmids play in the dissemination of antibiotic resistance genes, especially against carbapenems, in Mexican hospitals.

## Introduction

The genus *Acinetobacter* includes a group of bacteria that can be isolated from a wide variety of environmental sources, including soil and water. However, some of them have become important nosocomial pathogens, such as those included within the *Acinetobacter calcoaceticus-baumannii* complex. Members of this genus have been associated with severe nosocomial and community infections with high mortality rates. Nevertheless, isolates of other non-*baumannii* spp. have gained medical relevance because of their increased frequency in recent years, for example, *Acinetobacter haemolyticus*, *Acinetobacter lwoffii*, *Acinetobacter ursingii*, *Acinetobacter parvus*, and *Acinetobacter junii*.^1–3^

*Acinetobacter* spp. have developed resistance to multiple classes of antimicrobial agents, including broad-spectrum cephalosporins, carbapenems, fluoroquinolones, and aminoglycosides. This resistance is due to multiple mechanisms, such as resistance-nodulation-cell division (RND)-type efflux pumps, CarO porin, and resistance genes. In addition, the ability of *Acinetobacter* spp. to acquire mobilizable elements that carry antibiotic resistance genes increases the resistance dissemination.^4–6^ In particular, *bla*_NDM-1_, which encodes the New Delhi Metallo-β-lactamase-1 (NDM-1), hydrolyzes a broad spectrum of β-lactam antibiotics, including carbapenems, and is among the most worrisome resistance determinants that have spread around the world, severely complicating the treatment of nosocomial infections.^7–10^ In Latin America, its presence has been reported in Enterobacteriaceae isolates such as *Klebsiella pneumoniae*, *Escherichia coli*, and *Providencia rettgeri*,^11–13^ and in nonfermentative bacilli, such as *A. baumannii*, *Acinetobacter pittii*, *Acinetobacter bereziniae*, and *A. haemolyticus*.^14–17^ In this work, we report the presence of diverse antibiotic resistance genes in a strain of *A. haemolyticus* obtained from a Mexican pediatric patient and the characterization of the complete plasmid carrying *bla*_NDM-1_ isolated from this strain.

## Materials and Methods

### Bacterial isolation

*Acinetobacter haemolyticus* AN54 was recovered from peritoneal dialysis fluid culture from a 12-year-old male patient who had been admitted to the hospital for end-stage renal disease in February 2016. The patient was previously treated with ceftriaxone (CRO) and trimethoprim-sulfamethoxazole (SXT). The isolate was identified with the VITEK 2 system (bioMérieux) and molecular typing by sequencing of the *rpoB* gene.^18,19^

### Antimicrobial susceptibility testing

An antimicrobial susceptibility test was performed by using the agar disk diffusion method according to the Clinical and Laboratory Standards Institute guidelines.^20^ The following antimicrobials were tested: piperacillin (PIP), ticarcillin (TIC), ampicillin/sulbactam, piperacillin/tazobactam, ticarcillin-clavulanic acid (TIM), ceftazidime (CAZ), cefepime (FEP), cefotaxime (CTX), CRO, imipenem (IPM), meropenem (MEM), gentamicin, amikacin (AN), tetracycline, ciprofloxacin, levofloxacin, and SXT. The minimal inhibitory concentration (MIC) for CTX, CAZ, FEP, MEM, IPM, and AN was determined by the agar dilution method.^20^ Metallo-β-lactamase (MBL) detection was performed by IPM and MEM disks supplemented with 10 μL of 0.5 M ethylenediaminetetraacetic acid.^21^

### Test of the activity of the efflux pump in antibiotic resistance

The activity of the efflux pump was evaluated by using phenylalanine-arginine β-naphthylamide (Sigma-Aldrich) as an efflux pump inhibitor (EPI). The test was performed as follows: MICs for AN, CTX, and MEM were determined by the agar dilution method in the presence and absence of EPI (25 mg/L). A twofold or greater decrease in MIC in the presence of EPI was considered indicative of a role of RND-type efflux pumps in the resistance to the antibiotics tested. Test veracity was checked by using the strain *Acinetobacter haemolyticus* HNP11 as a positive control. To evaluate the effect of EPI on bacterial growth, all bacteria were cultured in Mueller–Hinton broth with and without EPI (25 mg/L).^22^

### Whole-genome sequencing and data analysis

A high-quality draft genome sequence from isolate AN54 was obtained by using an Illumina MiSeq platform (2 × 300 paired-end reads) (IBT-UNAM) and one SMRT cell of PacBio RS II system (Yale Center for Genome Analysis). With data obtained from both platforms, a hybrid assembly was performed with the Unicycler assembler version 0.4.1.^23^ and SPAdes version 3.11.1.^24^ ResFinder 2.1^25^ was used to identify and determine the location of antibiotic resistance genes. MAUVE version 20150226,^26^ CLC Sequence Viewer 8.0 and BLAST were used to align and compare sequences. EASYFIG 2.2.2 was used to draw figures.^27^

### Pulsed-field gel electrophoresis and Southern blot

The S1 nuclease-pulsed-field gel electrophoresis (PFGE) method was carried out to determine the plasmids number by using the *Escherichia coli* strain NCTC 50192 as a reference. To detect the resistance gene in the plasmid, the PFGE gel was transferred to a nylon membrane (Hybond-N; GE Healthcare Life Sciences), and hybridization was performed with the Dig-High Prime DNA Labeling and Detection Starter Kit II (Roche).

### Conjugation assays

Conjugation assays from AN54 to recipient strains *Escherichia coli* C600 (rifampicin resistant) and *Escherichia coli* DH5α (nalidixic acid resistant) were performed. Mueller–Hinton agar plates (BD Bioxon) supplemented with rifampicin (100 μg/mL) or nalidixic acid (32 μg/mL) containing AN (32 μg/mL) and MEM (8 μg/mL) were used for the selection of transconjugant strains.

#### Nucleotide sequence accession numbers

Draft genome and plasmid sequences of the AN54 strain were deposited in the GenBank database, with accession numbers CP041224.1 to CP041229.1.

## Results

In this work, we report the identification of an *Acinetobacter haemolyticus* (AN54) strain resistant to carbapenems, which was isolated from peritoneal dialysis fluid. This strain was initially identified as *Acinetobacter* spp. by a VITEK 2 System and subsequently reclassified as *A. haemolyticus* by analysis of the *rpoB* gene. The AN54 strain exhibited resistance to AN, and to the broad-spectrum β-lactams antibiotics, except for TIM. The *bla*_NDM-1_ and *bla*_OXA-265_ were previously detected by PCR and sequencing. A twofold decrease in the MIC for AN and no change in the MICs for CTX and MEM in the presence of EPI were observed. In addition, the phenotypic test to detect MBL production was positive (Table 1).

**Table 1.** Antimicrobial Susceptibilities Test and Genotype of *Acinetobacter haemolyticus* AN54

			*MIC (μg/mL)*							
*Strain*	*Anatomical site*	*Resistance phenotype*	*CAZ*	*FEP*	*IPM*	*CTX (CTX + EPI)*	*MEM (MEM + EPI)*	*AN (AN + EPI)*	*Genotype^a^*	*Efflux pumps genes^a^*
AN54	Peritoneal dialysis fluid	PIP, TIC, SAM, TZP, CAZ, FEP, CTX, CRO, IPM, MEM, AN	>128	>128	>128	>128 (>128)	>128 (>128)	64 (32)	*bla*_NDM-1_, *bla*_OXA-265_, *aph*A6, *aac*-(6’)-Ig	*adeA*, *adeB*, *adeC*, *adeI*, *adeK*, *adeS*,^*b*^*adeR*,^*b*^*macA*, *macB*

Control strain *Acinetobacter haemolyticus* HNP11 MIC values: CTX: 16 μg/mL, CTX + EPI: 8 μg/mL; AN: 64 μg/mL, AN + EPI: 32 μg/mL.

^a^ Obtained by PCR and whole-genome sequencing analysis.

^b^ Regulators of AdeABC efflux pump.

AN, amikacin; CAZ, ceftazidime; CRO, ceftriaxone; CTX, cefotaxime; EPI, efflux pumps inhibitor (phenylalanine-arginine-β-naphthylamide) (PAβN) 25 mg/L; FEP, cefepime; IPM, imipenem; MEM, meropenem; MIC, minimal inhibitory concentration; PIP, piperacillin; SAM, ampicillin/sulbactam; TIC, ticarcillin; TZP, piperacillin/tazobactam.

**FIG. 1.**
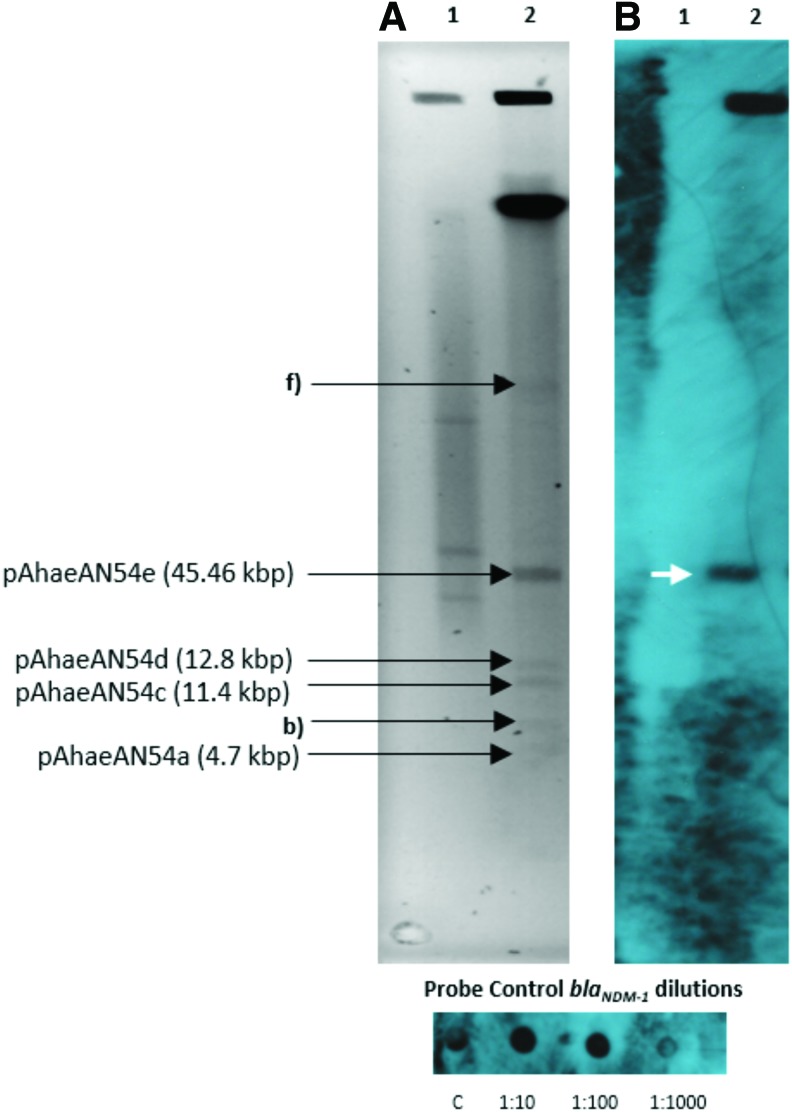
**(A)** PFGE-S1 gel showing the plasmids of *Acinetobacter haemolyticus* AN54 strain, Line 1: Control strain *Escherichia coli* NCTC 50192, Line 2: AN54. **(B)** Hybridization autoradiography with the *bla*_NDM-1_ probe (DIG-High Prime DNA Labeling and Detection Starter Kit II, Roche). The *white arrow* indicates that *bla*_NDM-1_ was detected in the plasmid pAhaeAN54e. The plasmid sizes were determined by WGS. PFGE, pulsed-field gel electrophoresis. Color images are available online.

Plasmid DNA extraction revealed that the AN54 isolate carries six different bands. Southern blot hybridization indicated that *bla*_NDM-1_ was present in one of the plasmids (Fig. 1).

Whole-genome sequencing analysis revealed that AN54 possess a chromosome with a size of ∼3.61 Mbp, and we obtained the complete sequence of four plasmids named pAhaeAN54a (4.7 kbp), pAhaeAN54c (11.4 kbp), and pAhaeAN54d (12.8 kbp) that do not carry antimicrobial resistance genes, and pAhaeAN54e (45.46 kbp) that carries *bla*_NDM-1_ and *aphA6*. In the chromosome, we detected the presence of *bla*_OXA-265_ and the aminoglycoside-modifying enzyme-encoding gene *aac(6’)-Ig*. In addition, genes for efflux pumps and their regulators that mediate multidrug resistance were found:, *adeA*, *adeB*, *adeC*, *adeI*, *adeK*, *adeR*, *adeS*, *macA*, and *macB*, as well as heavy metal resistance genes *czcA* and *arsH*.

The *bla*_NDM-1_ was located in the 45.46 kbp plasmid, which we named pAhaeAN54e. This plasmid has a 41% guanine-cytosine content and 53 open reading frames. Sixteen of these genes are related to plasmid maintenance and transfer functions (plasmid backbone) and include the plasmid-partitioning genes *parA* and *parB*, and transfer genes *traA*, *traC*, and *traD*, as well as some genes of the type IV secretion system (T4SS). Twelve genes of this plasmid are located within the composite transposon carrying *bla*_NDM-1_, and the remaining genes encode 25 hypothetical proteins. Conjugation assays were performed to test the transferability of this plasmid; however, no transconjugants were obtained under the tested conditions.

**FIG. 2.**
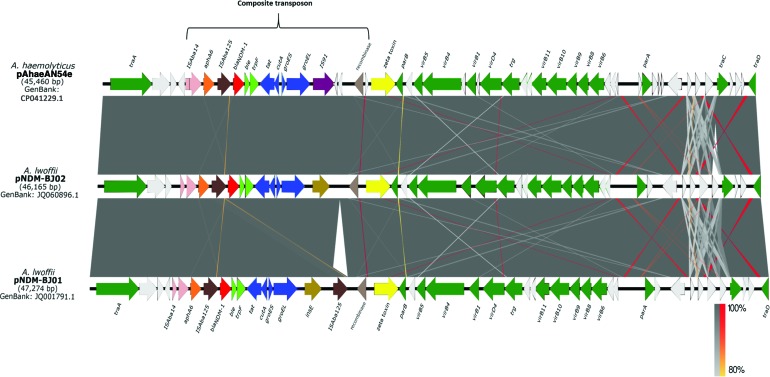
The schematic representation and alignment of plasmid pAhaeAN54e of *Acinetobacter haemolyticus* and plasmids pNDM-BJ02 and pNDM-BJ01 of *Acinetobacter lwoffii* are divided into two sections: the composite transposon in which IS*Aba125*, *bla*_NDM-1_, and IS*91* are inserted and the putative conjugation machinery. The known genes are marked in *green*, and putative proteins are marked in *white*. The *gray bars* indicate 99% identity, and the inverted regions are indicated in *red*. Color images are available online.

**FIG. 3.**
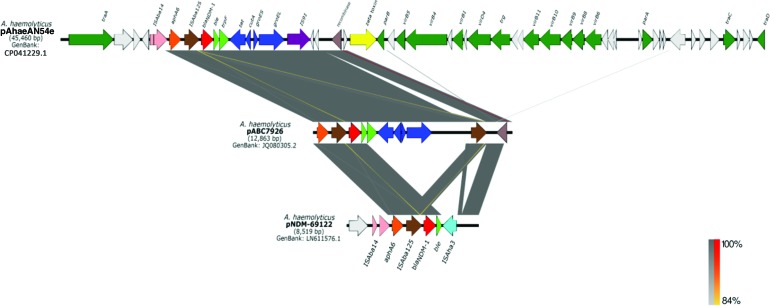
Comparison between the plasmid pAhaeAN54e and the partially sequenced plasmids pABC7926 and pNDM69122 of *Acinetobacter haemolyticus.* Color images are available online.

Plasmid pAhaeAN54e has 99% nucleotide identity, with 100% coverage, to plasmids pNDM-BJ02 (accession no. JQ060896.1) and pNDM-BJ01 (accession no. JQ001791.1) of *A. lwoffii*.^28^ Figure 2 shows the detected differences, especially the insertion of seven genes encoding hypothetical proteins along the T4SS gene cluster, near the *parA* and *traA* genes, and a deletion of a hypothetical protein upstream of *traC*. The pAhaeAN54e plasmid also has a truncated composite transposon that is similar to transposon Tn125. Both these transposons consist of the IS*Aba14* insertion sequence and the *aphA6*, *bla*_NDM-1_, *ble*, and *trpF* genes, but in contrast to Tn125, the truncated transposon of strain AN54 has only one copy of the insertion sequence IS*Aba125*. The structure of the truncated transposon is similar to that found in plasmid pNDM-BJ02 and to the partially sequenced plasmids pABC7926 (accession no. JQ080305.2) and pNDM-69122 (accession no. LN611576.1) of *A. haemolyticus* (Fig. 3).^29,30^

Plasmid pAhaeAN54e contains an IS*91* family transposase, which is 330 bp larger than the *ins*E transposase gene carried on the pNDM-BJ02 and pNDM-BJ01 plasmids in *A. lwoffii*. In addition, pAhaeAN54e has an insertion of two genes encoding hypothetical proteins downstream of IS*91* and one more that is adjacent to the recombinase gene.

**FIG. 4.**
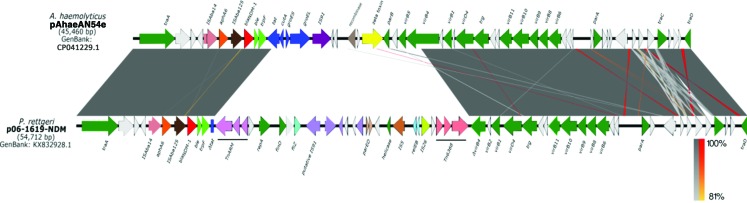
Comparison between plasmids pAhaeAN54e of *Acinetobacter haemolyticus* and p06-1619-NDM of *Providencia rettgeri*, both of which are isolated from México. Color images are available online.

Interestingly, plasmid pAhaeAN54e has 99% identity with more than 73% of coverage with the *P. rettgeri* plasmid p06-1619-NDM (accession no. KX832928.1) reported in Mexico (Fig. 4).^31^ These plasmids share 33 genes, including *traA*, *traC*, *traD*, and genes from the type IV secretion system. Plasmid p06-1619-NDM also has a Tn125-like element.

## Discussion

*A. baumannii* is one of the principal etiological agents causing nosocomial infections in Mexico and the rest of the world.^32–34^ In Mexican hospitals, there are a few reports of carbapenem-resistant *Acinetobacter* isolates that are not included in the *A. calcoaceticus*-*baumannii* complex,^17^ possibly because the molecular typing of these species is not routinely implemented in Mexican hospitals. In this work, we studied the resistome of an *A. haemolyticus* strain resistant to carbapenems. The strain carries different resistance mechanisms, such as *bla*_OXA-265_, which is a member of the *bla*_OXA-214_-like family on the *A. haemolyticus* chromosome.^35^ The strain also harbors aminoglycoside-modifying enzyme genes, such as *aac(6’)-Ig*, which is responsible for AN resistance and belongs to the AAC(6’)-I aminoglycoside N-acetyltransferase family reported in *Acinetobacter* spp.,^36,37^ and *aphA6*, which confers resistance to AN and is associated with the presence of *bla*_NDM-1_ in a plasmid from this strain, as has been reported in another study.^38^

Another antibiotic resistance mechanism studied in *A. baumannii* is the use of efflux pumps, mainly the RND family of efflux pumps, such as the AdeABC and AdeIJK systems, which are located on the bacterial chromosome. This family exhibits a wide substrate range that includes dyes, biocides, detergents, and antiseptics; however, its presence has been little studied in non-*baumannii* spp.^39^ The overexpression of this system was shown to be responsible for decreasing susceptibility to a broad spectrum of antimicrobials, such as aminoglycosides, tetracyclines, erythromycin, chloramphenicol, trimethoprim, fluoroquinolones, some β-lactams, and ethidium bromide, and has recently also been associated with tigecycline.^40^ The genes that encode the AdeABC efflux pump are organized in an operon (*adeABC*). There are two regulatory genes, *adeS* and *adeR*, and their products are closely related to proteins of the two-component regulatory system. These genes regulate efflux pump expression in response to cellular environment stimuli (antibiotics). This type of expression is called inductive.^41^

AdeIJK encoded by the *ade*IJK operon is the second RND efflux system described in *A. baumannii*,^42^ which contributes to the resistance to β-lactams, such as TIC, cephalosporins, aztreonam, fluoroquinolones, tetracyclines, tigecycline, lincosamides, rifampicin, and chloramphenicol; however, aminoglycosides are not substrates for this pump.^43^ The *Acinetobacter haemolyticus* AN54 strain contains homologues of the regulator proteins AdeS and AdeR and the efflux pump components AdeA, AdeB and AdeC with the *Acinetobacter baumannii* AYE strain, these proteins share an amino acid identity of 72%, 85%, 86%, 92% and 77% respectively, to the corresponding proteins. In addition, homologues of AdeI and AdeK proteins with 82% and 89% identity with *Acinetobacter baumannii* AYE were also found. However, the AdeJ subunit in strain AN54 was not located.

In this work, EPI was used to evaluate the role of the efflux pump in antibiotic resistance. The results obtained indicate that the efflux pump only participates in conferring resistance to AN, as shown by a twofold decrease in the MIC (from 64 to 32 μg/mL). The strain did not show a decrease in the MICs of CTX and MEM, possibly due to the presence of *bla*_NDM-1_.^6^

The presence of *bla*_NDM-1_ in a non-*baumannii* spp. of environmental origin such as *A. haemolyticus* is medically and epidemiologically relevant. This is especially true considering the lack of successful treatment for patients with some underlying diseases and the ease of *bla*_NDM-1_ dissemination through complex recombination events mediated by insertion sequences, transposons, and plasmids, as reported in previous works.^31,44,45^

The *Acinetobacter haemolyticus* AN54 strain harbors *bla*_NDM-1_ within a Tn125-like transposon in a plasmid highly similar to pNDM-BJ02 of *A. lwoffii*, which has been previously reported in China.^28^ The backbone of the plasmids carrying *bla*_NDM-1_ between *Acinetobacter* spp. is relatively conserved; however, one difference found in our plasmid is the presence of IS*91*. This gene is designated as IS*CR*, and one of its functions is the mobilization of additional sequences upstream of the transposase gene.^46,47^ The second difference is the insertion of seven genes encoding hypothetical proteins in the putative conjugation region; the function of these proteins is yet unknown. Currently, there are no methodologies to characterize this kind of replicon, due to the absence of a typical Rep protein, such as the one presented by Enterobacteriaceae plasmids, indicating that these plasmids possess an uncharacterized replication system; therefore, additional studies are needed. No transconjugants were obtained under the tested conditions. However, we cannot exclude other alternative mechanisms of plasmid transference, as reported in other studies of *Acinetobacter* spp. carrying *bla*_NDM-1_.^14,15,38,48^

Recently, Duran-Bedolla *et al*.^17^ partially reported the genetic context of *bla*_NDM-1_ in strains of *Acinetobacter* spp., including the *Acinetobacter haemolyticus* 10256 strain isolated in a different geographical area of Mexico. The plasmid reported in our work showed specific differences in comparison with the plasmid of *Acinetobacter haemolyticus* 10256 (55 kbp plasmid); these included size (45.4 kbp), absence of IS*Aba125* sequence in the 3′ end, presence of genes encoding hypothetical proteins along the structure of the plasmid, and IS*91* instead of IS*CR27*. These results suggest that the genetic context of *bla*_NDM-1_ in *A. haemolyticus* is partially conserved.^17,29,30^

Our study shows that plasmid pAhaeAN54e has a high similarity with plasmid p06-1619-NDM of *P. rettgeri* and, as noted by Marquez-Ortiz *et al.*,^31^ the similarities among pNDM-BJ01-like plasmids and plasmids of *P. rettgeri* may be the result of recombination events that lead to a chimeric plasmid that can be transmitted and replicated among Enterobacteriaceae and *Acinetobacter* isolates.^49^

## Concluding Remarks

In this study, we report a multidrug-resistant clinical *A. haemolyticus* strain isolated from a pediatric patient carrying *bla*_NDM-1_ in a novel variant of pNDM-BJ01-like plasmids. This plasmid could constitute a dissemination mechanism of antimicrobial resistance genes among diverse bacterial species. The findings reported in this work contribute to providing information about the diverse mechanisms of resistance that can coexist in *A. haemolyticus* strains.

## References

[1] Peleg A.Y., de Breij A., Adams M.D, Cerqueira G.M, Mocali S., Galardini M., Nibbering P.H, Earl A.M, Ward D.V, Paterson D.L, Seifert H., and Dijkshoorn L. 2012 The success of *Acinetobacter* species; genetic, metabolic and virulence attributes. PLoS One. 7:e46984 23144699 10.1371/journal.pone.0046984 PMC3483291

[2] Touchon M., Cury J., Yoon E.-J, Krizova L., Cerqueira G.C, Murphy C., Feldgarden M., Wortman J., Clermont D., Lambert T., Grillot-Courvalin C., Nemec A., Courvalin P., and Rocha E.P.C. 2014 The genomic diversification of the whole *Acinetobacter* genus: origins, mechanisms, and consequences. Genome Biol. Evol. 6:2866–2882 25313016 10.1093/gbe/evu225 PMC4224351

[3] Wong D., Nielsen T.B, Bonomo R.A, Pantapalangkoor P., Luna B., and Spellberg B. 2017 Clinical and pathophysiological overview of *Acinetobacter* infections: a century of challenges. Clin. Microbiol. Rev. 30:409–447 27974412 10.1128/CMR.00058-16 PMC5217799

[4] Esterly J., Richardson C.L, Eltoukhy N.S, Qi C., and Scheetz M.H. 2011 Genetic mechanisms of antimicrobial resistance of *Acinetobacter baumannii*. Ann. Pharmacother. 45:218–228 21304033 10.1345/aph.1P084

[5] Traglia G.M., Almuzara M., Vilacoba E., Tuduri A., Neumann G., Pallone E., Centrón D., and Ramírez M.S. 2014 Bacteremia caused by an *Acinetobacter junii* strain harboring class 1 integron and diverse DNA mobile elements. J. Infect. Dev. Ctries. 8:666–669 24820473 10.3855/jidc.3747

[6] Rumbo C., Gato E., López M., Ruiz de Alegría C., Fernández-Cuenca F., Martínez-Martínez L., Vila J., Pachón J., Cisneros J.M, Rodríguez-Baño J., Pascual A., Bou G., and Tomás M. 2013 Contribution of efflux pumps, porins, and β-lactamases to multidrug resistance in clinical isolates of *Acinetobacter baumannii*. Antimicrob. Agents Chemother. 57:5247–5257 23939894 10.1128/AAC.00730-13 PMC3811325

[7] Berrazeg M., Diene S.M, Medjahed L., Parola P., Drissi M., Raoult D., and Rolain J.M. 2014 New Delhi Metallo-beta-lactamase around the world: An eReview using Google Maps. Euro. Surveill. 19:pii: 10.2807/1560-7917.es2014.19.20.20809 24871756

[8] Shahid M. 2011 Environmental dissemination of NDM-1: time to act sensibly. Lancet Infect. Dis. 11:334–335 21478055 10.1016/S1473-3099(11)70074-3

[9] Roca I., Espinal P., Vila-Farrés X., and Vila J. 2012 The *Acinetobacter baumannii* oxymoron: commensal hospital Dweller turned pan-drug-resistant menace. Front. Microbiol. 3:148 22536199 10.3389/fmicb.2012.00148 PMC3333477

[10] Robledo I.E., Aquino E.E, Santé M.I, Santana J.L, Otero D.M, León C.F, and Vázquez G.J 2010 Detection of in KPC *Acinetobacter* spp. in Puerto Rico. Antimicrob. Agents Chemother. 54:1354–1357 20038618 10.1128/AAC.00899-09 PMC2825984

[11] Pasteran F., Albornoz E., Faccone D., Gomez S., Valenzuela C., Morales M., Estrada P., Valenzuela L., Matheu J., Guerriero L., Arbizu E., Calderon Y., Ramon-Pardo P., and Corso A. 2012 Emergence of NDM-1-producing *Klebsiella pneumoniae* in Guatemala. J. Antimicrob. Chemother. 67:1795–1797 22461309 10.1093/jac/dks101

[12] Torres-González P., Bobadilla-Del Valle M., Tovar-Calderón E., Leal-Vega F., Hernández-Cruz A., Martínez-Gamboa A., Niembro-Ortega M.D, Sifuentes-Osornio J., and Ponce-de-León A. 2015 Outbreak caused by Enterobacteriaceae harboring NDM-1 metallo-β-lactamase carried in an IncFII plasmid in a tertiary care hospital in Mexico City. Antimicrob. Agents Chemother. 59:7080–7083 26282410 10.1128/AAC.00055-15 PMC4604355

[13] Carvalho-Assef A.P.D., Pereira P.S, Albano R.M, Beriao G.C, Chagas T.P.G, Timm L.N, Da Silva R.C.F, Falci D.R, and Asensi M.D. 2013 Isolation of NDM-producing *Providencia rettgeri* in Brazil. J. Antimicrob. Chemother. 68:2956–2957 23869051 10.1093/jac/dkt298

[14] Pasteran F., Mora M.M, Albornoz E., Faccone D., Franco R., Ortellado J., Melgarejo N., Gomez S., Riquelme I., Matheu J., Ramon-Pardo P., and Corso A. 2014 Emergence of genetically unrelated NDM-1-producing *Acinetobacter pittii* strains in Paraguay. J. Antimicrob. Chemother. 69:2575–2578 24793901 10.1093/jac/dku139

[15] Brovedan M., Marchiaro P.M, Morán-Barrio J., Cameranesi M., Cera G., Rinaudo M., Viale A.M, and Limansky A.S. 2015 Complete sequence of a *bla*_(NDM-1)_-harboring plasmid in an *Acinetobacter bereziniae* clinical strain isolated in Argentina. Antimicrob. Agents Chemother. 59:6667–6669 26248354 10.1128/AAC.00367-15 PMC4576080

[16] Pillonetto M., Arend L., Vespero E.C, Pelisson M., Chagas T.P.G, Carvalho-Assef A.P.D, and Asensi M.D. 2014 First report of NDM-1-producing *Acinetobacter baumannii* sequence type 25 in Brazil. Antimicrob. Agents Chemother. 58:7592–7594 25288087 10.1128/AAC.03444-14 PMC4249496

[17] Duran-Bedolla J., Bocanegra-Ibarias P., Silva-Sánchez J., Garza-González E., Morfín-Otero R., Hernández-Castro R., Lozano L., Garza-Ramos U., and Barrios-Camacho H. 2018 Genetic characterization of multiple NDM-1-producing clinical isolates in Mexico. Diagn. Microbiol. Infect. Dis. 94:195–198 30642720 10.1016/j.diagmicrobio.2018.12.002

[18] Gundi V.A., Dijkshoorn L., Burignat S., Raoult D., and La Scola B. 2009 Validation of partial *rpoB* gene sequence analysis for the identification of clinically important and emerging *Acinetobacter* species. Microbiology. 155:2333–2341 19389786 10.1099/mic.0.026054-0

[19] La Scola B., Gundi V.A.K.B, Khamis A., and Raoult D. 2006 Sequencing of the *rpoB* gene and flanking spacers for molecular identification of *Acinetobacter* species. J. Clin. Microbiol. 44:827–832 16517861 10.1128/JCM.44.3.827-832.2006 PMC1393131

[20] Clinical Laboratory Standards Institute. 2017 Performance Standards for Antimicrobial Susceptibility Testing. Clinical and Laboratory Standards Institute, Wayne, PA

[21] Bonnin R.A., Naas T., Poirel L., and Nordmann P. 2012 Phenotypic, biochemical, and molecular techniques for detection of metallo-β-lactamase NDM in *Acinetobacter baumannii*. J. Clin. Microbiol. 50:1419–1421 22259204 10.1128/JCM.06276-11 PMC3318537

[22] López-García A., Rocha-Gracia R.C, Bello-López E., Juárez-Zelocualtecalt C., Sáenz Y., Castañeda-Lucio M., López-Pliego L., González-Vázquez M.C, Torres C., Ayala-Nuñez T., Jiménez-Flores G., de la Paz Arenas-Hernández M.M, and Lozano-Zarain P. 2018 Characterization of antimicrobial resistance mechanisms in carbapenem-resistant *Pseudomonas aeruginosa* carrying IMP variants recovered from a Mexican hospital. Infect. Drug Resist. 11:1523–1536 30288063 10.2147/IDR.S173455 PMC6160278

[23] Wick R.R., Judd L.M, Gorrie C.L, and Holt K.E. 2017 Unicycler: resolving bacterial genome assemblies from short and long sequencing reads. PLoS Comput. Biol. 13:e1005595 28594827 10.1371/journal.pcbi.1005595 PMC5481147

[24] Bankevich A., Nurk S., Antipov D., Gurevich A.A, Dvorkin M., Kulikov A.S, Lesin V.M, Nikolenko S.I, Pham S., Prjibelski A.D, Pyshkin A.V, Sirotkin A.V, Vyahhi N., Tesler G., Alekseyev M.A, and Pevzner P.A. 2012 SPAdes: a new genome assembly algorithm and its applications to single-cell sequencing. J. Comput. Biol. 19:455–477 22506599 10.1089/cmb.2012.0021 PMC3342519

[25] Zankari E., Hasman H., Cosentino S., Vestergaard M., Rasmussen S., Lund O., Aarestrup F.M, and Larsen M.V. 2012 Identification of acquired antimicrobial resistance genes. J. Antimicrob. Chemother. 67:2640–2644 22782487 10.1093/jac/dks261 PMC3468078

[26] Darling A.C.E., Mau B., Blattner F.R, and Perna N.T. 2004 Mauve: multiple alignment of conserved genomic sequence with rearrangements. Genome Res. 14:1394–1403 15231754 10.1101/gr.2289704 PMC442156

[27] Sullivan M.J., Petty N.K, and Beatson S.A. 2011 Easyfig: a genome comparison visualizer. Bioinformatics. 27:1009–1010 21278367 10.1093/bioinformatics/btr039 PMC3065679

[28] Hu H., Hu Y., Pan Y., Liang H., Wang H., Wang X., Hao Q., Yang X., Yang X., Xiao X., Luan C., Yang Y., Cui Y., Yang R., Gao G.F, Song Y., and Zhu B. 2012 Novel Plasmid and its variant haboring both a *bla*_NDM-1_ gene and type IV secretion System in clinical isolates of *Acinetobacrer lwoffii*. Antimicrob. Agents Chemother. 56:1698–1702 22290961 10.1128/AAC.06199-11 PMC3318331

[29] Jones L.S., Carvalho M.J, Toleman M.A, White P.L, Connor T.R, Mushtaq A., Weeks J.L, Kumarasamy K.K, Raven K.E, Török M.E, Peacock S.J, Howe R.A, and Walsh T.R. 2015 Characterization of plasmids in extensively drug-resistant *Acinetobacter* strains isolated in India and Pakistan. Antimicrob. Agents Chemother. 59:923–929 25421466 10.1128/AAC.03242-14 PMC4335910

[30] Fu Y., Liu L., Li X., Chen Y., Jiang Y., Wang Y., Yu Y., and Xie X. 2015 Spread of a common *bla*_NDM-1_-carrying plasmid among diverse *Acinetobacter* species. Infect. Genet. Evol. 32:30–33 25726900 10.1016/j.meegid.2015.02.020

[31] Marquez-Ortiz R.A., Haggerty L., Olarte N., Duarte C., Garza-Ramos U., Silva-Sanchez J., Castro B.E, Sim E.M, Beltran M., Moncada M.V, Valderrama A., Castellanos J.E, Charles I.G, Vanegas N., Escobar-Perez J., and Petty N.K. 2017 Genomic epidemiology of NDM-1-encoding plasmids in Latin American clinical isolates reveals insights into the evolution of multidrug resistance. Genome Biol. Evol. 9:1725–1741 28854628 10.1093/gbe/evx115 PMC5554438

[32] Bocanegra-Ibarias P., Peña-López C., Camacho-Ortiz A., Llaca-Díaz J., Silva-Sánchez J., Barrios H., Garza-Ramos U., Rodríguez-Flores A.M, and Garza-González E. 2015 Genetic characterisation of drug resistance and clonal dynamics of *Acinetobacter baumannii* in a hospital setting in Mexico. Int. J. Antimicrob. Agents. 45:309–313 25561030 10.1016/j.ijantimicag.2014.10.022

[33] Garza-González E., Llaca-Díaz J.M, Bosques-Padilla F.J, and González G.M. 2010 Prevalence of multidrug-resistant bacteria at a tertiary-care teaching hospital in Mexico: Special focus on *Acinetobacter baumannii*. Chemotherapy. 56:275–279 20693798 10.1159/000319903

[34] Morfín-Otero R., Alcántar-Curiel M.D, Rocha M.J, Alpuche-Aranda C.M, Santos-Preciado J.I, Gayosso-Vázquez C., Araiza-Navarro J.R, Flores-Vaca M., Esparza-Ahumada S., González-Díaz E., Pérez-Gómez H.R, and Rodríguez-Noriega E. 2013 *Acinetobacter baumannii* infections in a tertiary care hospital in mexico over the past 13 years. Chemotherapy. 59:57–65 23839011 10.1159/000351098

[35] Figueiredo S., Bonnin R.A, Poirel L., Duranteau J., and Nordmann P. 2012 Identification of the naturally occurring genes encoding carbapenem-hydrolysing oxacillinases from *Acinetobacter haemolyticus*, *Acinetobacter johnsonii*, and *Acinetobacter calcoaceticus*. Clin. Microbiol. Infect. 18:907–913 22128805 10.1111/j.1469-0691.2011.03708.x

[36] Doi Y., Wachino J.-I, Yamane K., Shibata N., Yagi T., Shibayama K., Kato H., and Arakawa Y. 2004 Spread of novel aminoglycoside resistance gene *aac(6’)-Iad* among *Acinetobacter* clinical isolates in Japan. Antimicrob. Agents Chemother. 48:2075–2080 15155202 10.1128/AAC.48.6.2075-2080.2004 PMC415623

[37] Lambert T., Gerbaud G., Galimand M., and Courvalin P. 1993 Characterization of *Acinetobacter haemolyticus aac(6’)-Ig* gene encoding an aminoglycoside 6’-N-acetyltransferase which modifies amikacin. Antimicrob. Agents Chemother. 37:2093–2100 8257129 10.1128/aac.37.10.2093 PMC192234

[38] Fu Y., Du X., Ji J., Chen Y., Jiang Y., and Yu Y. 2012 Epidemiological characteristics and genetic structure of *bla*_NDM-1_ in non-*baumannii Acinetobacter* spp. in China. J. Antimicrob. Chemother. 67:2114–2122 22604448 10.1093/jac/dks192

[39] Chu Y.W., Chau S.L, and Houang E.T.S. 2006 Presence of active efflux systems AdeABC, AdeDE and AdeXYZ in different *Acinetobacter* genomic DNA groups. J. Med. Microbiol. 55:477–478 16534000 10.1099/jmm.0.46433-0

[40] Gholami M., Hashemi A., Hakemi-Vala M., Goudarzi H., and Hallajzadeh M. 2015 Efflux pump inhibitor phenylalanine-arginine B-naphthylamide effect on the minimum inhibitory concentration of imipenem in *Acinetobacter baumannii* strains isolated from hospitalized patients in Shahid Motahari Burn Hospital, Tehran, Iran. Jundishapur. J. Microbiol. 8:e19048 26568800 10.5812/jjm.19048 PMC4639875

[41] Wieczorek P., Sacha P., Hauschild T., Zórawski M., Krawczyk M., and Tryniszewska E. 2008 Multidrug resistant *Acinetobacter baumannii*—the role of AdeABC (RND family) efflux pump in resistance to antibiotics. Folia Histochem. Cytobiol. 46:257–267 19056528 10.2478/v10042-008-0056-x

[42] Damier-Piolle L., Magnet S., Brémont S., Lambert T., and Courvalin P. 2008 AdeIJK, a resistance-nodulation-cell division pump effluxing multiple antibiotics in *Acinetobacter baumannii*. Antimicrob. Agents Chemother. 52:557–562 18086852 10.1128/AAC.00732-07 PMC2224764

[43] Coyne S., Guigon G., Courvalin P., and Périchon B. 2010 Screening and quantification of the expression of antibiotic resistance genes in *Acinetobacter baumannii* with a microarray. Antimicrob. Agents Chemother. 54:333–340 19884373 10.1128/AAC.01037-09 PMC2798560

[44] Al Atrouni A., Joly-Guillou M.-L, Hamze M., and Kempf M. 2016 Reservoirs of Non-*baumannii Acinetobacter* Species. Front. Microbiol. 7:49 26870013 10.3389/fmicb.2016.00049 PMC4740782

[45] Nordmann P., Poirel L., Walsh T.R, and Livermore D.M. 2011 The emerging NDM carbapenemases. Trends Microbiol. 19:588–595 22078325 10.1016/j.tim.2011.09.005

[46] Toleman M.A., Bennett P.M, and Walsh T.R. 2006 ISCR elements: novel gene-capturing systems of the 21st century? Microbiol. Mol. Biol. Rev. 70:296–316 10.1128/MMBR.00048-05 PMC1489542 16760305

[47] Toleman M.A., and Walsh T.R. 2010 ISCR elements are key players in IncA/C plasmid evolution. Antimicrob. Agents Chemother. 54:3534 20634542 10.1128/AAC.00383-10 PMC2916298

[48] Montaña S., Cittadini R., Del Castillo M., Uong S., Lazzaro T., Almuzara M., Barberis C., Vay C., and Ramírez M.S. 2016 Presence of New Delhi metallo-β-lactamase gene (NDM-1) in a clinical isolate of *Acinetobacter junii* in Argentina. New Microbes New Infect. 11:43–44 27257491 10.1016/j.nmni.2016.02.008 PMC4877398

[49] Barrios H., Garza-Ramos U., Reyna-Flores F., Sanchez-Perez A., Rojas-Moreno T., Garza-Gonzalez E., Llaca-Diaz J.M, Camacho-Ortiz A., Guzman-Lopez S., and Silva-Sanchez J. 2013 Isolation of carbapenem-resistant NDM-1-positive *Providencia rettgeri* in Mexico. J. Antimicrob. Chemother. 68:1934–1936 23620464 10.1093/jac/dkt124

